# Importance of interindividual interactions in eco‐evolutionary population dynamics: The rise of demo‐genetic agent‐based models

**DOI:** 10.1111/eva.13508

**Published:** 2022-11-27

**Authors:** Amaïa Lamarins, Victor Fririon, Dorinda Folio, Camille Vernier, Léa Daupagne, Jacques Labonne, Mathieu Buoro, François Lefèvre, Cyril Piou, Sylvie Oddou‐Muratorio

**Affiliations:** ^1^ E2S UPPA, INRAE, ECOBIOP Université de Pau et des Pays de l'Adour Saint‐Pée‐sur‐Nivelle France; ^2^ Management of Diadromous Fish in their Environment, OFB, INRAE, Institut Agro Univ Pau & Pays Adour/E2S UPPA Rennes France; ^3^ INRAE, UR 629 Ecologie des Forêts Méditerranéennes, URFM Avignon France; ^4^ CIRAD, UMR CBGP, INRAE, IRD, Montpellier SupAgro Univ. Montpellier Montpellier France

**Keywords:** agent‐based models, demo‐genetic models, DG‐ABMs, eco‐evolutionary dynamics, eco‐genetic models

## Abstract

The study of eco‐evolutionary dynamics, that is of the intertwinning between ecological and evolutionary processes when they occur at comparable time scales, is of growing interest in the current context of global change. However, many eco‐evolutionary studies overlook the role of interindividual interactions, which are hard to predict and yet central to selective values. Here, we aimed at putting forward models that simulate interindividual interactions in an eco‐evolutionary framework: the demo‐genetic agent‐based models (DG‐ABMs). Being demo‐genetic, DG‐ABMs consider the feedback loop between ecological and evolutionary processes. Being agent‐based, DG‐ABMs follow populations of interacting individuals with sets of traits that vary among the individuals. We argue that the ability of DG‐ABMs to take into account the genetic heterogeneity—that affects individual decisions/traits related to local and instantaneous conditions—differentiates them from analytical models, another type of model largely used by evolutionary biologists to investigate eco‐evolutionary feedback loops. Based on the review of studies employing DG‐ABMs and explicitly or implicitly accounting for competitive, cooperative or reproductive interactions, we illustrate that DG‐ABMs are particularly relevant for the exploration of fundamental, yet pressing, questions in evolutionary ecology across various levels of organization. By jointly modelling the effects of management practices and other eco‐evolutionary processes on interindividual interactions and population dynamics, DG‐ABMs are also effective prospective and decision support tools to evaluate the short‐ and long‐term evolutionary costs and benefits of management strategies and to assess potential trade‐offs. Finally, we provide a list of the recent practical advances of the ABM community that should facilitate the development of DG‐ABMs.

## 1 INTRODUCTION

Understanding and anticipating populations' response to changes in environmental and anthropogenic pressures requires conceptual and modelling approaches coupling ecological and evolutionary processes. This is largely motivated by the increasing realization that ecological and evolutionary responses of populations can occur on similar temporal scales, with potential consequences on dynamics from gene to ecosystem (Carroll et al., 2007). The burgeoning literature investigating eco‐evolutionary dynamics illustrates this growing interest (Bassar et al., 2021; Dunlop et al., 2009; Oddou‐Muratorio et al., 2020; Romero‐Mujalli et al., 2019; Schoener, 2011).

The conceptual framework of eco‐evolutionary dynamics depicts feedback loops between response processes at different levels of biological organization in a contemporary timescale (Govaert et al., 2019; Hendry, 2017; Pelletier et al., 2009). These feedback loops acknowledge that (1) genetic diversity and its architecture determine the demographic structure and population dynamics through phenotypic expression; (2) demographic structure and population dynamics determine evolutionary processes, that is genetic drift, selection and gene flow, which in turn (3) determine genetic diversity. As an illustration of such feedback, the competition between trees within a forest results in a selection process contributing to genetic evolution, while the genetic composition of the tree population drives interindividual competition and forest productivity (Pretzsch, 2021). To account for feedback loops, eco‐evolutionary models must integrate inheritance mechanisms and the multiple driving forces controlling the dynamics of the distributions of heritable traits across generations (Bassar et al., 2021).

One of these key drivers of selection is the interactions between individuals within populations, as they directly or indirectly affect individual fitness at the core of any evolutionary dynamics (Maynard Smith, 1974; Webber & Vander Wal, 2018). We focus here on *within*‐population interindividual interactions (i.e. competition, cooperation and mating) affecting the demographic dynamics (growth, reproduction and mortality) and ultimately individual fitness or even inclusive fitness (Box 1). In essence, the outcome of such interactions is eminently stochastic and context‐dependent, and population structure itself is part of the context. It is now recognized that the structure of social networks within a population may affect natural selection and trait evolution through indirect genetic effects (traits affected by genes in other individuals, Fisher & McAdam, 2017; Kazancioǧlu et al., 2012; Marjanovic et al., 2022; Wade et al., 2010). Additionally, these networks are themselves dynamic, since changing the social environment may influence an individual's later decisions in a social interaction, leading to rapid shifts in networks' structures (Farine & Whitehead, 2015). For instance, individuals are able to modify their mating tactics, which diminishes the selection they endure (Oh & Badyaev, 2010) and thus affects selection at the population level. Likewise, the distribution of phenological traits (e.g. flowering or maturation time) shapes mating opportunities within plant and animal populations and possibly leads to assortative mating (here, the positive correlation of phenology between mates). Compared with random mating, assortative mating can either deplete or increase the genetic variance available for selection depending on whether the environment is stable or changing, with contrasted consequences on genetic adaptation (Godineau et al., 2021). Unfortunately, the interindividual interactions are usually little appreciated in eco‐evolutionary models, with potential consequences on our understanding of the full range of eco‐evolutionary responses.

**BOX 1:** Interindividual interactions involved in population eco‐evolutionary dynamics

Here, we focus on interactions between conspecific individuals within a population—mainly competition, cooperation, and mating—which directly drive the processes of mortality, growth, and reproduction (e.g. Figure A, C, D below) and whose variations subsequently induce evolutionary changes. This also includes the variety of ecological interactions indirectly impacting demography, such as exchange of information (e.g. on predator, or resource availability), movement (e.g. to escape predation or competition) or group behaviour (e.g. affecting predator's avoidance or resistance, Figure A, B below).
The major reason why we focus on local (i.e. within‐population), variable, conspecific interactions is that evolution is a population‐specific process, primarily fuelled by differences in individual fitness arising from the response to abiotic and biotic environments, the latter including the social context. Interspecific interactions may also shape the within‐population social context and contribute to evolution: for instance, the existence and strength of plant‐pollinator interactions define the social context within which selfing may evolve (Katsuhara et al., 2021). Trophic interactions may contribute to the resource context within which functional traits related to resource acquisition may evolve (Kang & Thibert‐Plante, 2017). On a macroevolutionary timescale, intra‐ and inter‐specific competition for resources can drive speciation (Gavrilets et al., 2007; Weber et al., 2017). However, considering interspecific interactions without genetic variation in at least one of the partners of the interaction is not enough to model the dynamic feedback loop between ecological interactions, fitness, and the genetic composition of the population. This is particularly why predation was not considered as a focal interaction in this review: indeed, when predation is investigated from the point of view of the variation of a prey's trait conferring variable avoidance ability from the predator, or from the variation of a predator's trait conferring variable ability to catch prey, then it becomes a trait involved in competition among prey to escape predators, or among predators to optimize prey foraging and selection (e.g. Kelly & Phillips, 2019; Labonne & Hendry, 2010). 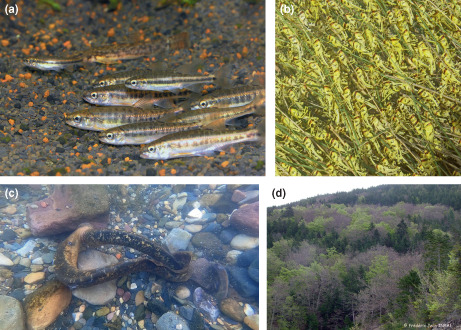
(a) School of common minnow (*Phoxinus phoxinus*) individuals maintained in an experimental tank at INRAE, Saint‐Pée‐sur‐Nivelle, France. Schooling behaviour in this species is supposed to be both an anti‐predator and a foraging optimisation strategy (Photo: ©INRAE—Stéphane Glise)
(b) Fifth instar hoppers of gregarious desert locust basking in the morning sun within herbaceous plants of the Mauritanian desert; grouping behaviours and bright coloration in desert locust (*Schistocerca gregaria*) are supposed to be an anti‐predator strategy (Photo: ©JIRCAS—Koutaro Ould Maeno)
(c) Sea lamprey (*Petromyzon marinus*) spawning in the Nive River (South‐western France). Species from the Petromyzontidae family are semelparous, but the number of mates is highly variable among species (Photo: ©INRAE—Stéphane Glise)
(d) Beech (*Fagus sylvatica*) trees with late and early phenologies on Mont‐Ventoux, France. Phenological mismatch limits male more than female reproductive success (Photo: ©INRAE—Frédéric Jean)

Our objectives here are to put forward models that explicitly *or* implicitly account for variable within‐population interindividual interactions in an eco‐evolutionary framework: the demo‐genetic agent‐based models (DG‐ABMs). After defining these models, we survey the literature to illustrate how DG‐ABMs can be used to investigate fundamental issues in evolutionary ecology, as well as to assist the management of natural populations facing environmental changes.

## 2 HOW TO MODEL ECO‐EVOLUTIONARY FEEDBACK LOOPS: FROM ANALYTICAL MODELS TO DG‐ABMS

**FIGURE 1:**
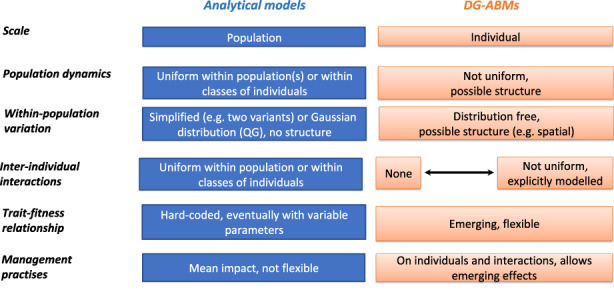
Different approaches to model eco‐evolutionary feedback loops. This scheme summarizes the main differences between two major modelling approaches used to investigate eco‐evolutionary dynamics: analytical models on the left and DG‐ABMs on the right. Their main difference is that analytical models consider evolutionary and/or ecological processes to be homogeneous within groups of individuals (the population or life‐stages), whereas DG‐ABMs can account for phenotypic and genotypic variation in groups of individuals, its effects on individual decisions/traits linked to local and instant conditions, and their outcome at the group level (i.e. emerging effects). In particular, some (although not all) DG‐ABMs model interindividual interactions, and their effects on individual fitness, which emerge in part from these interactions. QG, quantitative genetics.

At the very core of the eco‐evolutionary models is the need of specifying the genetically variable and heritable traits, their impact on the focal organism's life history and the ecological embedding that determines how life‐history traits affect and are affected by environmental conditions and the demographic context (Dieckmann & Ferriere, 2004). This can be achieved by various approaches (Figure 1). First, there is a long tradition in evolutionary ecology to rely on analytical models (differential‐equation and difference equation models), which offer elegant solutions and provide general knowledge on elementary eco‐evolutionary feedback loops, generally at the cost of simplifying hypotheses. Among the most common analytical formalisms of eco‐evolutionary feedback loops are (1) adaptive dynamics models (Dieckmann & Ferriere, 2004), which incorporate ecological realism, in particular, the notion that the success of any given strategy depends on its frequency within the population, but often bypass the complexity of genotype–phenotype relationship (for instance by assuming asexual reproduction, clonal inheritance); (2) evolutionary quantitative genetics models (Kirkpatrick & Barton, 1997; Pease et al., 1989; Slatkin, 1978), which integrate the genotype–phenotype map with population demography (e.g. density‐dependence) but where other ecological changes remain independent from the population dynamics; and (3) integral projection models (Smallegange & Coulson, 2013), which use population models classically developed in population dynamics to describe the evolution of continuous characters in a quantitative genetics framework. We purposely do not mention traditional optimisation models, such as stochastic dynamic programming used to represent individual behaviour (e.g. life‐history decisions) and development (e.g. growth and sexual maturity) and their consequences for population dynamics (Mangel, 2015), as these models do not specify the genetic architecture of traits, which is yet mandatory for eco‐evolutionary feedback to emerge. The main limitation of the above‐listed analytical approaches is that they consider evolutionary and ecological processes (be they deterministic or stochastic) to be homogeneous within groups of individuals (the population or life stages), whereas group composition constantly varies in terms of phenotypes and genotypes, affecting individual decisions, linked to local and instant conditions, and their outcome at the group level (i.e. emerging effects).

Yet the question of individual heterogeneity and its effects has long preoccupied eco‐evolutionary ecologists. For several decades, simulations using agent‐based models (ABMs, also called individual‐based models or IBMs in ecology) were used to investigate more complex scenarios and explore unexpected eco‐evolutionary feedback loops, with approaches spreading on a spectrum of complexity well‐described by DeAngelis and Mooij (2005). On the one side of the spectrum, some ABMs were developed to validate and/or explore the predictions made with analytical models, replace these models and/or eventually nurture their future development. To keep these ABMs as simple as possible, individuals usually have a minimum number of attributes and fitness does not depend on interindividual interactions. For instance, by coupling a niche‐based model with individual‐based demo‐genetic simulations, Cotto et al. (2020) investigated the evolutionary constraints related to alpine plant response to a changing climate. The key originality of their approach is to model individuals as spatial points across a complex climatic landscape, where the individual phenotypes are explicitly linked to climatic variables and where the optimal phenotype is prescribed by the niche‐based model and varies through time. They use a classical multistage life cycle model (from seeds to adults) where individual survival and ultimately fitness increases when the multivariate phenotype is close to the optimal phenotype but is independent of the phenotype of other individuals. This typical top‐down approach aims at extending classic analytical models into more complex domains with the assistance of ABMs.

On the other side of the spectrum, some ABMs employ a specific bottom‐up approach to fully integrate individual interactions and their outcome over time and space within a population, the result of which will dictate the strength and direction of evolutionary processes at the population level (DeAngelis & Mooij, 2005; Huston et al., 1988). These ABMs acknowledge that individuals have inherently nonuniform interactions with each other, and that the consequences of the variation in traits mediating interindividual interactions are better described by rule‐based simulations than by mathematical models. Accordingly, these approaches depict the interactions between individuals and their effects on individual fitness, accounting for the social context, and observe the resulting dynamics in terms of distributions of heritable traits and demography. We hereafter refer to these ABMs as DG‐ABMs, DG‐ABMs (another possible acronym would be eco‐genetic ABMs).

DG‐ABMs can be defined as individual‐based (meta) population dynamics models with heritable trait variation and phenotype‐dependent interactions between individuals (Box 2). A key feature of DG‐ABMs is that fitness variation emerges mechanically from interactions between individuals (as opposed to assuming an a priori fitness function) and gives rise to the evolution of patterns structuring the population diversity and its dynamics (e.g. genetic architecture and spatial genetic structure). Typical examples of emerging fitness variation are spatially structured individual‐based models focussing on dispersal evolution (Bach et al., 2006; Kubisch et al., 2013; Poethke et al., 2007). Indeed, these studies demonstrated that genetic structure and kin competition emerge from the spatial design of their DG‐ABMs, when the genetic architecture of dispersal and competition is included (here implicitly). Hence, dispersal evolves to reduce kin competition and increase inclusive fitness, ultimately driving back kin structure within populations. This is radically different from assuming a prescribed relationship between traits and fitness, as done in analytical models and some ABMs (e.g. Cotto et al., 2020). We argue here that this bottom‐up construction of fitness in DG‐ABMs provides different and new insights into various fundamental and applied questions in ecology and evolution, and illustrate further our point of view by a review of the literature.

**BOX 2:** An overview of demo‐genetic agent‐based models (DG‐ABMs), and on how they model interindividual interactions

*Conceptual scheme of DG‐ABMs (Figure below)*. Individuals (or agents) are characterized by their phenotypic traits, determined by their genotype, the environment, and interactions between them (denoted G × E). The agents together define the population, hence determining its diversity and structure, where interindividual interactions shape the social environment. This social environment influences population dynamics, which ultimately drives evolutionary processes (drift, selection, gene flow). Fitness variations (e.g. survival, fecundity variation) emerge from different outcomes of interindividual interactions (e.g. mating, competition, cooperation, information exchange) and give rise to evolution of traits via the trans‐generational response to selection. This framework, highlighting the feedback loop central to eco‐evolutionary approaches, is the core part of DG‐ABMs and is identified by solid (units)/dashed (units' properties) line boxes and bold arrows 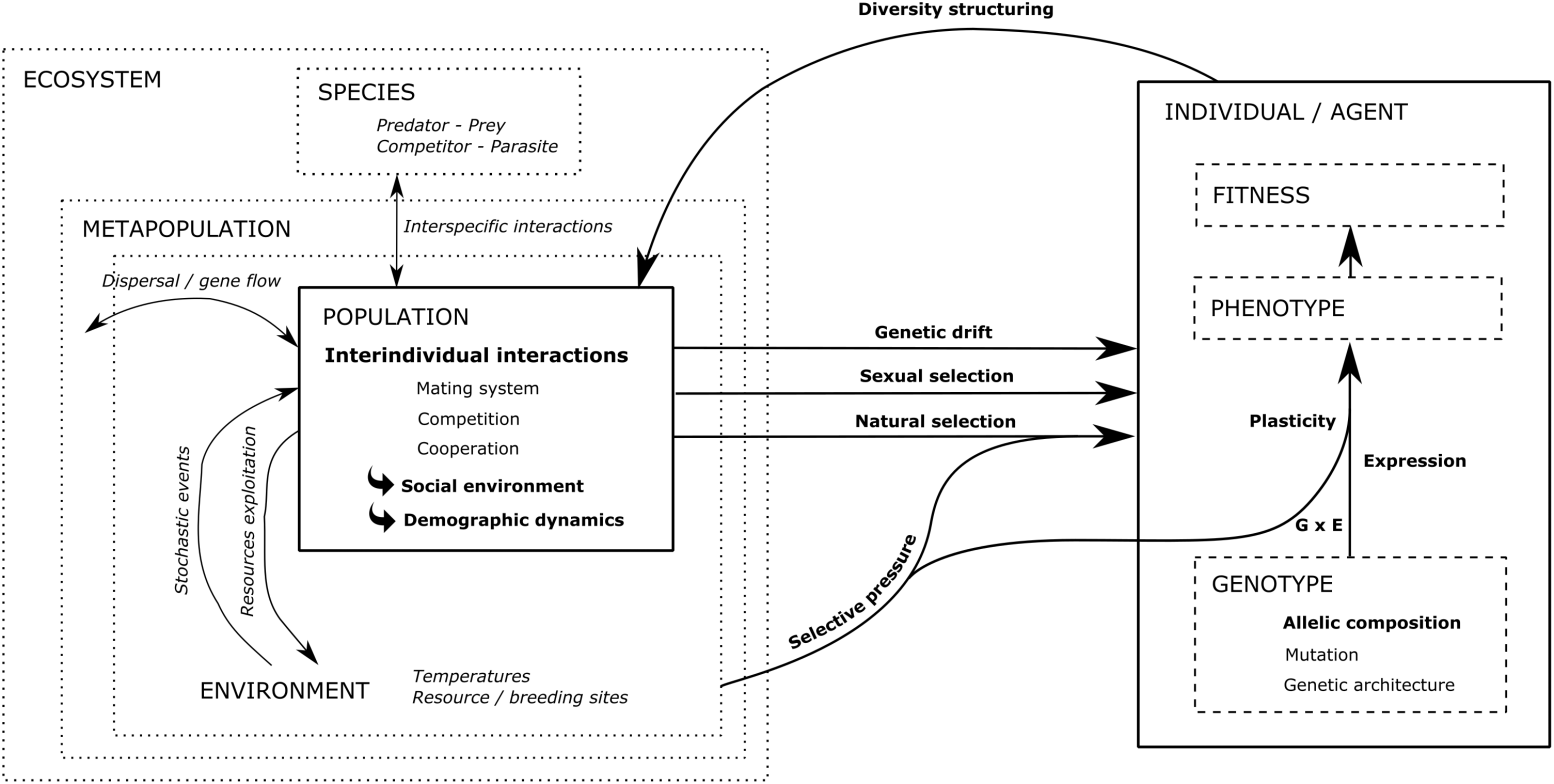
*Modelling interindividual interactions*: ABMs have the general capacity to represent both *direct* interactions among agents (i.e. when one agent identifies one or more other agents and directly affects them, for example by having some kind of contest with them, eating them, or choosing them to mate) and *mediated/indirect* interactions (when one agent affects others indirectly by producing or consuming a shared resource)
The choice to model these interactions *explicitly or implicitly* in DG‐ABMs depends on the interaction type, the degree of realism/complexity desired, and on the focal, evolvable trait(s) involved in the interaction (see Table 1 for examples of these traits). Direct reproductive interactions are most often explicitly modelled, through variable mate preference or competitiveness among potential mates (e.g. Chevalier et al., 2022), or assortative mating for a variable phenological trait (e.g. Soularue & Kremer, 2014). This is also the case of direct cooperative interactions, where the mechanisms involved (e.g. in grouping behaviour) are usually explicitly represented (de Jager et al., 2020; Van Der Post et al., 2015)
Indirect interactions such as competition for resources can be implicitly modelled through density‐dependence functions. For instance, most DG‐ABMs investigating fisheries‐induced evolution assume that increasing density will lead to increasing competition, the competition strength also depending on individual size (Ivan & Höök, 2015; Piou & Prévost, 2012). By contrast, some DG‐ABMs consider competition in an explicit prey–predator (Costa et al., 2016) or consumer‐resource (Kang & Thibert‐Plante, 2017) system; in these cases, the level of the resource and the consumption process at each time step are explicitly modelled, and the traits involved in the interaction can be more realistically represented (e.g. gill‐raker count in Kang & Thibert‐Plante, 2017)
*DG‐ABMs applications*: DG‐ABMs also offer the opportunity to study eco‐evolutionary dynamics at multiple levels of organization and spatio‐temporal scales. At population scale, habitat structuring and variation in the abiotic environment can be included to account for selection, stochastic events and subdivision of the social environment. These models also allow simulations of several populations' dynamics connected through dispersal with potential gene flow, such as in a metapopulation case. At a higher level, community dynamics can be modelled through interspecific interactions between individuals from directly or indirectly interacting species

## 3 OBJECTIVE AND METHOD FOR THE LITERATURE REVIEW

In their recent review of individual‐based modelling of eco‐evolutionary dynamics, Romero‐Mujalli et al. (2019) illustrated how ABMs have been applied to assess organisms' and populations' responses to environmental change, but overlooked whether these ABMs accounted or not for interindividual interactions. Here, we specifically reviewed DG‐ABMs in which fitness variation emerges mechanically from interactions between individuals.

To that aim, we searched the Web of Science Core Collection between 1955 and 2022 for various combinations of keywords (Figure S1). A first query using (Individual‐based model* OR IBM*) AND (eco‐evol* OR demo‐genet* OR demogenet* OR ecogenet* OR eco‐genet*) returned 138 publications. Using the terms (Agent‐based model OR ABM) instead of (Individual‐based model* OR IBM*), we obtained 15 publications indicating that the eco‐evolutionary community has not appropriated the term ABM despite its broader meaning (e.g. Railsback & Grimm, 2019). Of all these 153 publications (Table S1), only 54 included the terms ([interindivid* OR inter‐individ* OR individ*] AND interact*). After excluding reviews, technical publications, book chapters, preprint and duplicated studies (Table S2), we retained 120 publications. Finally, as we were interested in studies using a DG‐ABM approach, we checked whether these 120 remaining publications (1) use an IBM; (2) simulate dynamics over multiple generations; (3) represent (direct or indirect) interactions between conspecific individuals; (4) represent individual variation in the interaction‐related trait(s); and (5) consider that part of this variation is heritable. With this method, we filtered out 45 additional publications that did not satisfy these five criteria, resulting in a total of 75 publications using DG‐ABMs where interindividual interactions affect fitness. Using a nonexhaustive snowball approach, we found 14 additional references cited in or citing the 75 selected publications (see Table S3 and Lamarins et al., 2022 for the final database). Note that the difficulties we encountered in selecting studies using DG‐ABMs with interindividual interactions from the WOS illustrate the need for clearer referencing based on keywords better shared by the community.

## 4 SYNTHESIS OF THE LITERATURE REVIEW

In the selected 89 studies, competition was by far the most considered interaction (79 studies), followed by reproductive interactions (38 studies) and cooperative interactions (four studies only). We found 32 studies accounting for two types of interaction simultaneously.

On average, 1.9 traits (between 1 and 19 traits) per study were considered as evolvable. The nature of evolvable trait(s) depended on the interaction type, the species/kingdom considered and the level of generality/realism/precision of the model (following the classification of models properties of Levins, 1966). We distinguished eight categories of evolvable traits (Tables 1 and 2): (1) traits related to growth and/or maturation thresholds (36 studies); (2) traits related to mating (12 studies); (3) dispersal traits (12 studies); (4) traits related to cognitive behaviour and information exchange (six studies); (5) traits related to energy acquisition or allocation (six studies); traits related to (6) defence (five studies) or (7) virulence (two studies); (8) and finally, abstract traits—meaning that they do not correspond directly to a measurable trait—generally related to competitive ability or/and assortative mating (17 studies). We found seven studies considering two types of traits simultaneously. While some of these traits directly mediate interindividual interactions (e.g. mating traits for reproduction, behavioural traits for cooperation), most of them indirectly impact interactions. For instance, dispersal traits or movement preferences are often associated with avoidance of competition and/or predation, or mate search for reproduction (Fronhofer & Altermatt, 2017; Travis et al., 2012). Traits related to growth, maturation and energy acquisition or allocation, influence individual size, which often plays a major role in the outcome of competition.

**TABLE 1:** Interindividual interactions and associated evolvable traits modelled in DG‐ABMs

IT	Evolvable traits		Examples of references (species/kingdom)
	Category	Examples	
Competition	(1) growth/maturation	Size at emergence	Fielding (2004) (grasshopper); Ayllón et al. (2016, 2018) (trout)
		Threshold for size at migration	Piou and Prévost (2012, 2013) (salmon)
		Growth rate	Kang and Thibert‐Plante (2017) (alewife); Moya‐Laraño (2011) (generic); Travis et al. (2010) (plant)
	(2) abstract trait	Competitive abilities	Gascuel et al. (2015), Pontarp et al. (2015), Ward and Collins (2022) (all generic for species community)
	(4) dispersal trait	Prospecting of habitat quality	Fronhofer and Altermatt (2017), Ponchon et al. (2021) (generic)
		Dispersal distance	LaRue et al. (2019) (sea rocket); Leidinger et al. (2021) (plant)
	(5) behaviour	Movement preference	Hrycik et al. (2019) (perch)
		Drifting	Mazzucco et al. (2015) (shrimps)
	(6) energy, allocation	Functional traits related to energy acquisition	Ivan and Höök (2015) (perch); Mollet et al. (2016) (plaice)
	(7) defence	Toxin production	de la Peña et al. (2011) (plant‐herbivores)
		Abstract defence	Costa et al. (2016), Urban et al. (2019) (generic)
	(8) virulence	Pathogen virulence	Papaïx et al. (2018), Rimbaud et al. (2018) (plant pathogen)
REPRODUCTION	(1) growth/maturation	Threshold for size at maturity	Ayllón et al. (2016, 2018) (trout); Piou and Prévost (2012, 2013) (salmon)
		Slope/intercept of the maturation reaction norm	Dunlop et al. (2007) (bass)
	(3) mating	Selfing or self‐incompatibility	Kirchner et al. (2006), Katsuhara et al. (2021) (plant)
		Mate choice (preference, competitiveness), mate search	Berec et al. (2018), Chevalier et al. (2022) (generic); Labonne and Hendry (2010) (guppy); Nathan et al. (2019) (trout)
Cooperation	(2) abstract trait	Mutualistic or antagonistic trait	Maliet et al. (2020) (generic)
	(5) cognitive behaviour	Grouping, schooling behaviour	Van Der Post et al. (2015) (generic); Reuter et al. (2016) (fish)
		Attachment density	de Jager et al. (2020) (mussel)

*Note*: To illustrate the categories of traits considered as evolvable in the reviewed DG‐ABMs, we listed some examples depending on the interaction type considered (IT).

**TABLE 2:** Association between the category of evolvable traits considered in each DG‐ABM, and the type of eco‐evolutionary feedback considered

Trait category	Type of eco‐evolutionary feedback					
	Ecology‐focussed	Microevolution‐focussed	Macroevolution‐focussed	Management‐focussed	Spatial‐focussed	Number of studies
Growth/Maturation	6	2	3	16	3	29
Abstract trait	2	7	8			17
Dispersal	2				9	11
Mating	2	5		1		8
Cognitive behaviour	2	1	1		2	6
Defence	1	2			1	4
Energy acquisition or allocation	2	1		1		4
Virulence				2		2
Mating & growth/mat.			1	2		3
Mating & Energy acq) or allocat.		1				1
Growth/ mat. & Defence				1		1
Growth/mat. & Dispersal					1	1
Growth/mat. & Energy acq. or allocat.				1		1
Number of studies	17	19	13	24	16	89

These evolvable traits are at the core of the eco‐evolutionary feedback loops in DG‐ABMs, since fitness variation emerges from interactions among individuals that differ in these traits, giving rise to population dynamics in terms of both distribution of evolvable traits and demography. We distinguished five main types of eco‐evolutionary feedback in the reviewed DG‐ABMs (Table 2). We found 17 ‘Ecology‐focussed’ DG‐ABMs, with a high level of realism in the demographic and ecological processes, and incorporating a ‘dose’ of evolutionary processes to gain a better understanding of the ecological/demographic behaviour. In these DG‐ABMs, evolvable traits were most often growth/maturation traits, but six other trait categories were considered. Then, we found 19 ‘Microevolution‐focussed’ DG‐ABMs, with a high level of generality in the evolutionary processes, and incorporating a ‘dose’ of demographic and ecological processes to gain a better understanding of the evolutionary behaviour at a contemporary timescale. Similarly, there were also 13 ‘Macroevolution‐ focussed’ DG‐ABMs, dedicated to the understanding of speciation at a macroevolutionary timescale. In these ‘Micro‐ or macroevolution‐focussed’ DG‐ABMs, the evolvable trait was most often abstract, but mating traits were also often considered. Then, we identified 24 ‘Management‐ focussed’ DG‐ABMs, used to address how management practices interfere with eco‐evolutionary feedbacks; in these DG‐ABMs, evolvable traits were most often growth/maturation traits. Finally, we found 16 ‘Spatial‐focussed’ DG‐ABMs, used to investigate eco‐evolutionary feedback loops in a spatially explicit context (e.g. metapopulation). These DG‐ABMs investigated in particular the evolution of dispersal traits.

Another characteristic of DG‐ABMs is the type of inheritance framework used to model genetic variation in the evolvable traits. We found that 64 studies (71.9%) used a Mendelian inheritance process either in a population genetic framework (one locus, possibly multi‐allelic, which directly determines the phenotype) or combined with a quantitative genetic framework (several loci, together with the environment, which govern trait variation). Besides, 22 studies (24.7%) used an infinitesimal quantitative genetic framework (where each offspring inherits the mean of the two parent's genetic values), and two studies (2.2%) tested for population versus quantitative genetic framework. Note that our definition of DG‐ABM is larger than the one suggested by some authors (e.g. Frank & Baret, 2013), who proposed to reserve the term ‘eco‐genetic’ to models based on a quantitative genetics framework, and the term ‘demo‐genetic’ to models based on a population genetics framework.

Beyond these general typologies, we illustrate below the main applications of the reviewed DG‐ABMs, through selected examples.

## 5 DG‐ABMS TO BETTER UNDERSTAND ECO‐EVOLUTIONARY FEEDBACK LOOPS

Accounting for variable within‐population interindividual interactions in a bottom‐up approach allows DG‐ABMs to better investigate the emergence of fitness variation resulting from several complex eco‐evolutionary processes and the interactions between them. Accounting for the stochastic and context‐dependent outcomes of competitive, cooperative or reproductive interactions can change the predicted evolution of life‐history traits compared with an approach where the relationship between traits and fitness is prescribed. Below, we emphasize relevant studies from our literature review which investigate these three types of interaction.

We start with examples of DG‐ABMs considering explicit *competitive* interactions within species. Fielding (2004) investigated competition in grasshoppers and showed that contrasted optimal values of life‐history traits can emerge from different types of localized interindividual interactions, that is exploitative or size‐based competition. In their DG‐ABM of trout population, Ayllón et al. (2016) observed the emergence of different eco‐evolutionary outcomes due to explicit competitive interactions for food in a changing environment. These two DG‐ABMs with explicit competitive interactions were built from well‐tested demographic models, and additionally considered that the same traits (size at emergence and maturity size threshold) could evolve and interact with the spatial distribution of food resources to shape population dynamics. Most often in the reviewed DG‐ABMs focussing on single species adaptive dynamics, competition is implicitly considered, for example through a density‐dependence function. In a perch species, Ivan and Höök (2015) showed variable patterns of energy allocation along individual ontogeny, resulting from the interplay between plastic and adaptive responses to selection and density‐dependent competition for food. Using a DG‐ABM representing competition among individuals choosing different life‐history tactics, Piou and Prévost (2012, 2013) showed that climate change may modify salmon population dynamics through plastic responses of individual size. These two DG‐ABMs acknowledge the main role of individual size on competition, and incorporate both genetic and plastic variation into this trait to gain a better understanding of the adaptive population dynamics in future, changing environments.

Integrating behavioural interactions between individuals and eco‐evolutionary feedback is logically critical to understand the evolution of sociality and *cooperation*. Van Der Post et al. (2015) investigated how grouping, a taxonomically widespread social process, co‐evolved with two cooperative social behaviours: anti‐predator vigilance and foraging. In a simulation experiment where behavioural processes were specified through 19 variable traits, but not the cost and benefits of each decision strategy, they showed eco‐evolutionary interactions between group size and vigilance with an evolutionary trajectory towards bigger groups and less vigilance, eventually leading to fission into small groups with high vigilance and back. Accounting for heritable interindividual differences and environmental heterogeneity in resource distribution, Reuter et al. (2016) were able to relate landscape structuration to the evolution of schooling behaviour and collective foraging in fish. Although these studies mostly focussed on how cooperation can emerge in models where costs and benefits are not explicitly specified but related to other behavioural traits, reverse strategy, where cooperation is the evolvable trait, could also be used to investigate adaptive dynamics.

*Reproductive interactions* are an obvious major driver of demographic dynamics, and ‘Ecology‐focussed’ DG‐ABMs are particularly suitable to investigate this issue in an eco‐evolutionary framework. For instance, to explore how mating behaviour and population size jointly affect fitness components or population growth rate through Allee effects, Berec et al. (2018) considered the rate of mate search as evolvable and found different optimal values of search rates for populations at different densities, resulting in lower Allee thresholds in populations kept at lower densities. DG‐ABMs are also relevant to examine the interplay between demographic processes and the mating system when self‐incompatibility (Kirchner et al., 2006) or sterility (Nonaka & Kaitala, 2020) occur as a direct consequence of the genotype.

*Reproductive interactions* are also known to drive evolutionary dynamics (Maan & Seehausen, 2011), and explicit representation of mating interactions is important as sexual selection can sometimes oppose natural selection (Labonne & Hendry, 2010), or eventually reinforce it (Soularue & Kremer, 2014). Mate choice strongly depends on the population structure, making the outcome challenging to predict yet rarely random (Klug & Stone, 2021). DG‐ABMs, by allowing to represent explicitly sexual interactions, are particularly adapted to explore the evolution of traits considering the dynamic aspects of mating systems, such as when sexual preference and competition over mating partners occur, while still accounting for natural selection (Chevalier et al., 2022; Nathan et al., 2019). In this context, growth traits, or traits related to life‐history decisions such as migration or maturation, are often chosen as key traits to jointly consider size‐dependent survival and reproductive interaction and their possible interactions (Ayllón et al., 2019; Piou et al., 2015). Another application is the investigation of sexual dimorphism, which can arise when a given trait is subject to different selection pressures in males versus females (or even opposing pressures in the case of sexual conflict), but has a shared genetic basis between the sexes. Höök et al. (2021) showed how sex‐specific plasticity for size could evolve by looking at perch evolutionary response to environment. Kane et al. (2022) showed that optimal migration propensity differed among males and females in trout, and that populations could adapt to environmental change across a range of intersex genetic correlations for migration propensity, which influence the magnitude of sexual conflict.

## 6 EXTENDING IN SPACE, TIME AND LEVELS OF ORGANIZATION

In most examples detailed above, eco‐evolutionary dynamics are modelled within a non‐spatially explicit population. However, the spatial arrangement of habitats shapes animal movements or gametes propagation, and therefore also shapes social interactions and sexual networks (He et al., 2019). Since they allow fine‐scale explicit representation of habitats as well as individual movements, DG‐ABMs are well‐suited to represent *spatial evolutionary dynamics*. Focussing on the evolution of dispersal, Fronhofer and Altermatt (2017) showed how eco‐evolutionary feedback can emerge from a simple spatially explicit DG‐ABM. Depending on network topology and connectivity, variable evolutionary stable dispersal strategies emerged from their model via kin competition, and lead to eco‐evolutionary feedback by changing back the network's demography and genetic structure. Hrycik et al. (2019) explored the importance of environmental cues in perch vertical movement. By allowing movement rules in response to these cues to evolve, they illustrated the role of DG‐ABMs in determining appropriate movement rules in spatially explicit ecological modelling. Travis et al. (2010) used a mechanistic DG‐ABM approach to model the evolution of seed dispersal in plant populations, accounting for likely trade‐offs between traits in a patchy landscape. Additionally, sexual selection can determine the reproductive success of immigrants in populations and thus the strength and direction of demo‐genetic consequences of dispersal (e.g. demographic rescue, evolutionary rescue vs. gene swamping). For instance, Soularue and Kremer (2014) highlighted the major importance of gene flow and assortative mating in shaping the genetic differentiation between populations in a heterogeneous environment.

Interactions between conspecific individuals are at the core of DG‐ABMs. In addition, considering explicitly higher levels of organization (e.g. community level) to represent *interspecific interactions* may ultimately change the evolutionary outcomes expected from single species systems (Terhorst et al., 2018; Weber et al., 2017). We found examples of such multispecies DG‐ABMs used to investigate mating interactions: for instance, using an ABM in which two plant species share the same pollinators, Katsuhara et al. (2021) highlighted that the evolution of selfing without pollinator assistance (autonomous selfing) may increase population growth rates of inferior competitors and consequently favour long‐term coexistence via an evolutionary rescue. Furthermore, McDonald et al. (2019) showed that the strength of intraspecific competition for mates may result from sexual interactions with heterospecifics, which may interfere with sexual selection (i.e. interspecific reproductive interference).

Most of the reviewed multispecies DG‐ABMs focussed on competitive interactions, in an explicit prey–predators' or community context. For instance, Kang and Thibert‐Plante (2017) illustrated that considering trophic interactions and the genetic basis of functional traits within a single model could improve the understanding of evolutionary morphological changes in fish. Hillaert et al. (2020) showed that in a fragmented habitat, the presence of predators selects for increased herbivore movement and hence larger herbivore size. Demo‐genetic models of plant‐virus interactions allowed to investigate the emergence of plant viral genotypes breaking down plant qualitative resistance genes (Fabre et al., 2009). Ecological interactions at the community level may drive selection within species, and selection may affect in return the processes of species assembly at a community scale (Leidinger et al., 2021). Finally, as multispecies DG‐ABMs represent both intra‐ and interspecific complexity, they are especially suited to address macroevolutionary consequences of interspecific interactions, such as speciation (Gavrilets et al., 2007; Weber et al., 2017). We found several examples of macroevolution‐focussed DG‐ABMs developed to investigate adaptive radiation, that is the rapid diversification of a single lineage into many species with a great diversity of ecological strategies (Gascuel et al., 2015; Pontarp et al., 2015; Ward & Collins, 2022). These models generally consider a limited number of abstract, phenotypic traits reflecting the competitive ability of the focal individual with all the other individuals of the local patch. The distance between these ecological phenotypes within a patch drives exploitative competition, while heritable variation of the ecological phenotype fuels the processes of local adaptation and speciation.

Overall, it appears that DG‐ABMs have a large potential to address fundamental eco‐evolutionary questions accounting for multiple drivers of fitness, and are increasingly used in an integrative way, allowing effects to flow up and down between organization levels.

## 7 DG‐ABMS TO ASSIST MANAGEMENT STRATEGIES

Another key feature of DG‐ABMs is their capacity to model the effects of management practices on individuals and their interactions, together with that of other eco‐evolutionary processes. Hence, by allowing emerging effects, DG‐ABMs can also be efficient prospective tools to elaborate and assess management strategies. When management consists of *demographic control of populations*, in particular through individual phenotype‐based choices, it can deeply impact all demographic processes and population genetic composition, and therefore the intensity and direction of the evolutionary processes (Lefèvre et al., 2013). For example, selective fishing (or harvesting) directly affects competition among surviving fish (or trees), while genetic composition determines optimal fishing (or harvesting) patterns. In particular, different DG‐ABMs were used to understand how selective fishing can affect the demography and evolution of fish populations (fisheries‐induced evolution), through cascading and sometimes counterintuitive effects on population demographic structure, growth and maturation thresholds (Ayllón et al., 2018; Piou et al., 2015; Wang et al., 2017; Wang & Höök, 2009). By simultaneously modelling the plastic and genetic responses of individuals, DG‐ABMs can also disentangle the role of selective fishing and environment in the observed and predicted population declines and phenotypic changes (Piou et al., 2015).

When evolutionary dynamics and *land use planning decisions* are linked, DG‐ABMs also represent valuable decision support tools. For example, Papaïx et al. (2018) and Rimbaud et al. (2018) used a spatially explicit demo‐genetic model to assess the joint effect of crop cultivar deployment strategies in space and time and key pathogen life‐history traits on epidemiological dynamics, resistance durability and long‐term evolutionary control. Using a DG‐ABM, Mims et al. (2019) found strong effects of spatial connectivity on demo‐genetic outcomes in reintroduced bull trout populations, and allowed identification of watershed areas with higher persistence probabilities.

In the case of *hybridization* between native/wild and introduced/domesticated gene pools, DG‐ABMs allow to study the impact of management on the dynamics of crossing within and between gene pools, which depends on differential social interactions (e.g. mating preference) and genetic performances (e.g. local adaptation) between gene pools (Castellani et al., 2015; Nathan et al., 2019). In this context, DG‐ABMs are an effective means of developing genetic enrichment strategies in a prospective approach (which genetic resources and which deployment modalities for which risks?), and conversely of evaluating strategies aimed at preserving the local gene pool from unwanted introgression.

In these different case studies, DG‐ABMs offer a relevant framework to evaluate the short‐ and long‐term evolutionary costs and benefits of management actions and to assess potential trade‐offs between them. For example, they allow to address the issue of exploiting a population or a metapopulation (e.g. fishing and wood production) while preserving its genetic value and diversity, or to determine how to minimize the risks of demo‐genetic collapses of populations facing climate change. Furthermore, by controlling the social context of populations, management drives the overall ecological processes and thus affects biotic and abiotic stressors, the susceptibility of populations to these stressors, and selection intensity (Jactel et al., 2009).

## 8 TAKING ADVANTAGE OF ABMS FOR DG‐ABMS

The above‐listed examples from our literature review illustrate the diversity of interindividual interactions, adaptive traits and ecological processes that can be investigated using DG‐ABMs. This diversity is a strength but requires active strategies to better identify possible links between similar models developed to answer different questions, and to structure the community of developers and users of these models. Identified as ABMs, DG‐ABMs can benefit from multiple advances in the ABM community. The flexibility of the approach ranges from very simple and generic models to very complex and specific models, depending on model assumptions and objectives (Edmonds & Moss, 2005). A wide panel of tools and methodologies are available to explore DG‐ABMs (Thiele et al., 2014). The exponential increase in genomic databases should help in the calibration/validation of DG‐ABMs (Rudman et al., 2018). The use of description protocols such as Overview, Design concepts and Details protocol ensures the replicability and enhances the understanding of the models (Grimm et al., 2020). The TRACE framework (Grimm et al., 2014) is also a powerful tool for planning, documenting and assessing model development, analysis and application. Software for ABM development have increased in simplicity, quality, speed of computation and reliability and allow sharing pieces of code easily (Dufour‐Kowalski et al., 2012); in particular, quantitative genetic libraries can be plugged into existing population dynamic models to describe the genetic architecture of adaptive traits (e.g. ‘Genetics’ library in CAPSIS Dufour‐Kowalski et al., 2012; Oddou‐Muratorio & Davi, 2014). Software for complex model exploration have been proposed (Reuillon et al., 2013). Complex and multi‐authored models may use modelling notebooks to keep trace of all steps of conceptualisation, model development, implementation and exploration in order to enhance the confidence of end‐users of DG‐ABMs in the management communities (Ayllón et al., 2021). Finally, the publication of model codes on specific dissemination platforms is encouraged in the ABM community (e.g. https://www.comses.net/codebases/). All these recommendations should benefit the development of DG‐ABMs.

Intrinsically, DG‐ABMs conception requires a multidisciplinary approach integrating multiple levels of knowledge and can be used in interdisciplinary research projects as a tool of interaction among disciplines. ABMs are also used as frontier objects in several contexts (Le Page & Perrotton, 2017; Reilly et al., 2021). As such, DG‐ABMs are important tools in interacting with management or other end‐user communities that need to incorporate evolutionary processes in their decisions. Although this has not been done so far, DG‐ABMs could even be developed as part of a participatory modelling approach (Le Page et al., 2012) to integrate the knowledge of a diverse community of experts that need to manage constantly evolving ecosystems. Finally, they should become essential to adaptive management with an evolutionary perspective (Groot & Rossing, 2011).

## 9 CONCLUSION

In complement to the analytical models traditionally employed by evolutionary ecologists to investigate eco‐evolutionary feedback loops, this review puts forward DG‐ABMs, which are individual‐based (meta)population dynamics models with heritable trait variation and phenotype‐dependent interactions between individuals. Our literature review illustrates how the bottom‐up construction of fitness in these DG‐ABMs allows them to provide new insights into various fundamental and applied questions in ecology and evolution.

Previous reviews of the literature have indicated that ABMs in general are not used to address general questions in ecology and evolution, but have a more ‘narrow’ or ‘pragmatic’ scope (DeAngelis & Grimm, 2014). We advise modellers working on eco‐evolutionary processes to carefully consider the benefits of accounting for the effects of interactions between individuals on fitness in their approach, since it might significantly affect the direction and magnitude of evolution. This is true for theoretical investigations and for more applied objectives, since these eco‐evolutionary mechanisms also operate on rather short timescales (a handful of generations). Using a dedicated term—such as DG‐ABM—would facilitate a distinction between categories of modelling approaches, highlighting the specifics of eco‐evolutionary models accounting for interindividual interactions and their variations, and the potential differences in their respective predictions.

## CONFLICT OF INTEREST

The authors declare that they have no competing interests.

## Supporting information


Figure S1

Table S1

Table S2

Table S3

## Data Availability

The database of the 89 original research studies using DG‐ABMs with interindividual interactions affecting fitness is available at: https://doi.org/10.57745/FUQGSG.
