# Corrigendum

**DOI:** 10.1111/eva.13527

**Published:** 2023-01-18

In Evolutionary Applications 15:12, for the article by Lamarins et al. (2022) entitled “Importance of interindividual interactions in eco‐evolutionary population dynamics: The rise of demo‐genetic agent‐based models” (pages 1988–2001), the authors would like to include an Author Contributions section as follows:

## AUTHOR CONTRIBUTIONS

All authors contributed equally to the conceptualization of the ideas developed in this study, and to the initial review and synthesis of the literature. Hence, the first five authors (A.L., V.F., D.F., C.V. and L.D.) should be considered as co‐first authors, while the five last authors (J.L., M.B. F.L., C.P and S.O‐M.) should be considered as co‐senior authors. The doctoral students (A.L., V.F., D.F., C.V. and L.D.) wrote the first draft of this manuscript and designed the figures. A smaller group (A.L., L.D., M.B., F.L. and S.OM) took on the second literature survey required by the reviewers. All authors reviewed, edited and approved the final manuscript. J.L and C.P were involved in the acquisition of funds.
