# Supplementary material for: Importance of interindividual interactions in eco‐evolutionary population dynamics: The rise of demo‐genetic agent‐based models
Source: Evol Appl. 2022 Nov 27;15(12):1988–2001. doi: 10.1111/eva.13508 (PMC9753837; doi:10.1111/eva.13508)

Figure S1a: Distribution over time of the 138 selected publications associated to "IBM" and "eco-evolution" keywords. Keywords: (Individual-based model* OR IBM)* AND (eco-evol* OR demo-genet* OR demogenet* OR ecogenet* OR eco-genet*). WOS 1955/01/01-2022/07/01.

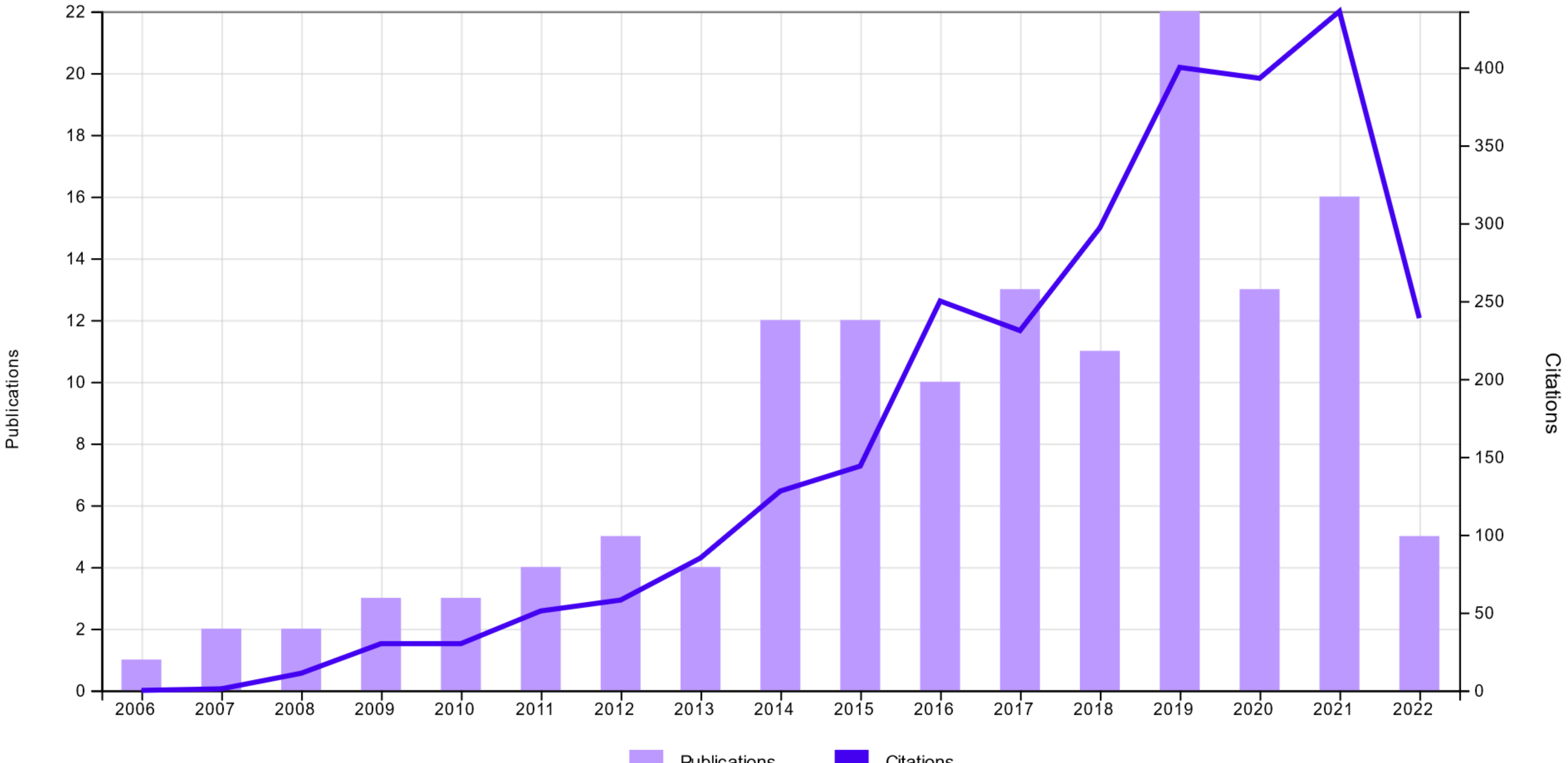

Figure S1b: WOS categories of the 138 selected publications associated to “IBM” and “eco-evolution” keywords. Keywords: (Individual-based model* OR IBM)* AND (eco-evol* OR demo-genet* OR demogenet* OR ecogenet* OR eco-genet*). WOS 1955/01/01-2022/07/01.

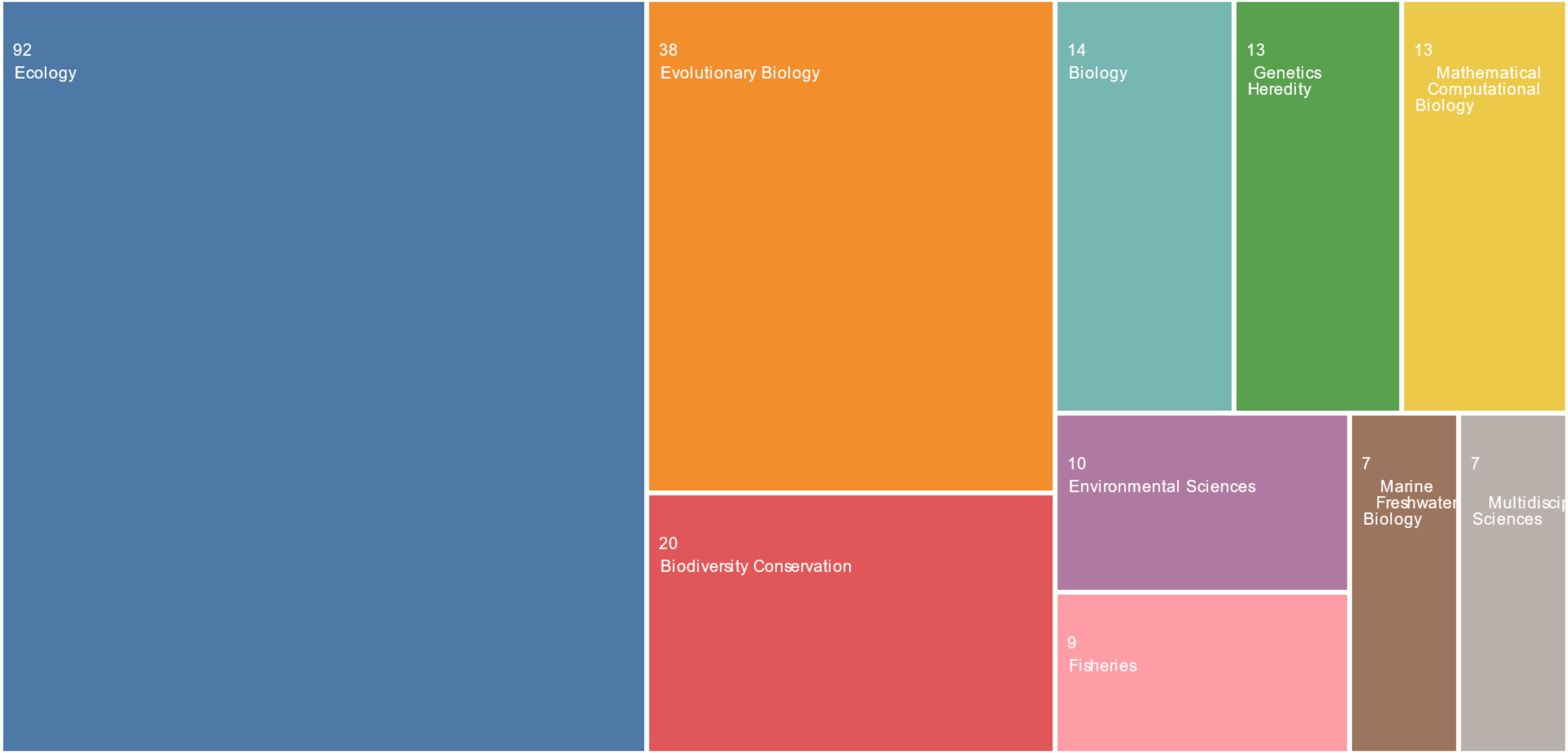

Figure S1c: Distribution over time of the 15 selected publications associated to “ABM” and “eco-evolution” keywords. Keywords: (Agent-based model* OR ABM)* AND (eco-evol* OR demo-genet* OR demogenet* OR ecogenet* OR eco-genet*). WOS 1955/01/01-2022/07/01.

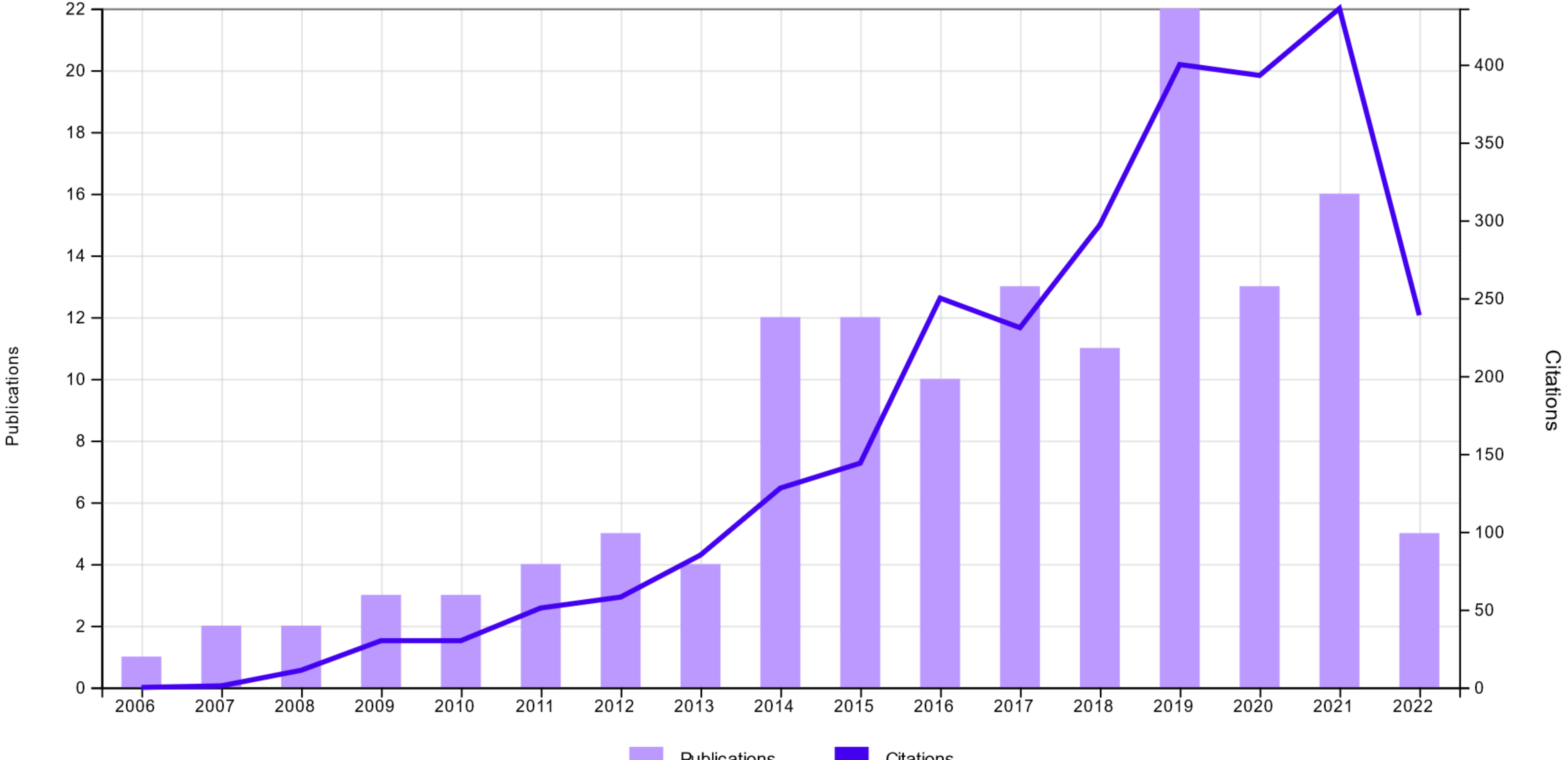

Figure S1d: WOS categories of the 15 selected publications associated to “ABM” and “eco-evolution” keywords. Keywords: (Agent-based model* OR ABM)* AND (eco-evol* OR demo-genet* OR demogenet* OR ecogenet* OR eco-genet*). WOS 1955/01/01-2022/07/01.

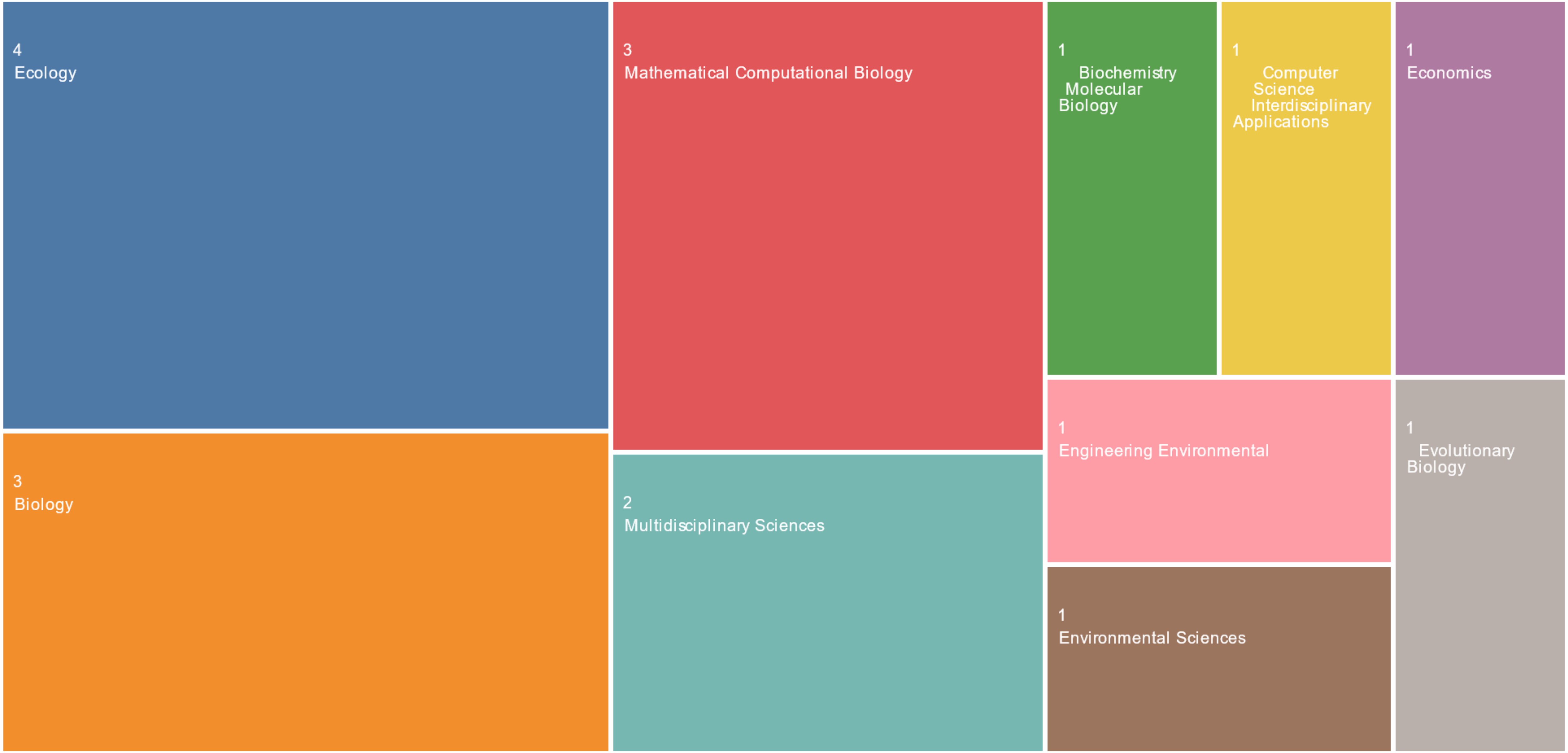

Supplement: Supplementary file 1 — Figure S1 [file EVA-15-1988-s003.pdf]
