# Supplementary material for: Importance of interindividual interactions in eco‐evolutionary population dynamics: The rise of demo‐genetic agent‐based models
Source: Evol Appl. 2022 Nov 27;15(12):1988–2001. doi: 10.1111/eva.13508 (PMC9753837; doi:10.1111/eva.13508)
Supplement: Supplementary file 3 — Table S2 [file EVA-15-1988-s002.docx]

**Table S2. Publications excluded during the literature review.**

This table focuses on original publications of models that were not retained. The sub-categories a) to d) correspond to other models than individual-based models (IBMs). The sub-category e) corresponds to IBMs applied on a time scale of maximum 1 generation, i.e., without evolution. The sub-category f) corresponds to IBMs which do not represent neither explicit nor implicit interactions between conspecific individuals. Finally, the sub-category g) corresponds to IBMs including interindividual interactions, but either without individual variation in the interaction-related trait(s), or with purely plastic variation in these traits.

The category “Not appropriate” gathers: 15 reviews, seven technical publications (introducing a new software without specifical investigation of an eco-evolutionary issue), five publications related to cell biology model (without any link to ecology), three book chapters, two duplicated studies, one conference proceeding, and one preprint.

| Category | Sub-Category | Number of publications |
| --- | --- | --- |
| 1- Original publication of a new model but… | a) Concept paper | 1 |
|  | b) Niche model | 1 |
|  | c) Differential equation model | 1 |
|  | d) Integral projection model (IPM) | 2 |
|  | e) IBMs without evolution | 7 |
|  | f) IBMs without interactions | 13 |
|  | g) IBMs with interactions but without eco-evolutionary feedback | 19 |
| 2- DG-ABM with interindividual interactions affecting fitness | | 75 |
| 3- Not appropriate (review, software, proceeding publication) or duplicates | | 34 |
| Total | | 153 |
